# Dr. (Miss) Rupa Bai Furdoonji: World’s first qualified lady anaesthetist

**DOI:** 10.4103/0019-5049.65371

**Published:** 2010

**Authors:** Narayana Ala, Bharathi K, Subhaktha PKJP, Manohar Gundeti, Ramachari A

**Affiliations:** 1 Director, National Institute of Indian Medical Heritage (Formerly known as Indian Institute of History of Medicine), 3^rd^ Floor Osmania Medical College, Hyderabad, India; 2,3 Assistant Director (Ay.), National Institute of Indian Medical Heritage (Formerly known as Indian Institute of History of Medicine), 3^rd^ Floor Osmania Medical College, Hyderabad, India; 4 Research Officer (Ay.), National Institute of Indian Medical Heritage (Formerly known as Indian Institute of History of Medicine), 3^rd^ Floor Osmania Medical College, Hyderabad, India; 5 Chief Anesthetist (Rtd.), ESIS Hospital, Hyderabad, India

## INTRODUCTION

Dr. (Miss) Rupa Bai Furdoonji was the first lady anaesthetist of the world. She had administered anaesthesia in the British residency hospital (present Sultan Bazaar hospital), Afzalgunz Hospital and Zenana Hospital, Hyderabad, in the years 1889-1917 A.D.

The chief surgeon of British residency, also the Principal of Hyderabad Medical School, Surgeon Major (IMS) Edward Lawrie has eulogised her expertise in administering Chloroform anaesthesia in page (274), in the book ‘A Report on Hyderabad Chloroform Commissions’ published in 1891 A.D.[1]

In those days, there was neither separate speciality like anaesthesiology nor a separate specialist like anaesthesiologist. Surgeons used to anaesthetise the patients and handover the unconscious patient to the care of a nurse or medical student. The expertise of Rupa Bai was highly appreciated. She was deputed to Edinburgh-U.K. in 1909 to gain more experience and knowledge about anaesthetics. As there was no separate qualification available in anaesthesia in those days, she obtained Diploma in Physics and Chemistry from Edinburgh University, because the knowledge of these subjects was found useful for the doctors who handled anaesthetics.

## GLIMPSES OF HER CAREER

**Figure 1:**
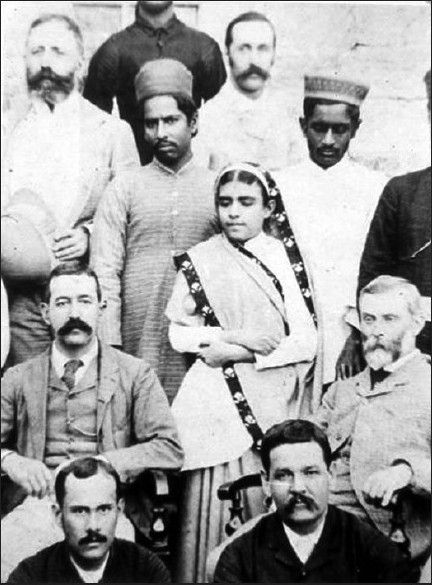
Miss Rupa Bai with Surgeon major Edward Lawrie (sitting on her right) and Sir Thomas Lauder Brunton, F.R.S. (sitting on her left)

Mr. Harmusji Kause, a Parsee gentleman of Hyderabad, gave me the glimpses of her career. I met him in the year 1988 A.D. This gentleman had preserved her original certificates and some important letters, pertaining to her career. They are preserved in the Indian Institute of History of Medicine in Osmania Medical College, Hyderabad. I am indebted to Mr. Harmsji because he could identify the individuals in the group photograph of second Hyderabad Chloroform Commission. The revelation that she could be the first lady anaesthesist of the world, occurred to me after going through the proceedings of second International conference of History of Anaesthesia in London in year 1987 A.D. All my efforts failed to trace her career after her retirement in about 1920 A.D. She retired from Nizam’s Medical service as superintendent of Chaderghat Hospital, also known as British residency Hospital (Sultan bazaar hospital) [Figure 1].

## HISTORICAL BACKGROUND

Hyderabad was the capital city of a state known as Nizam’s dominion in the southern peninsula of Indian subcontinent. Those were the days of British rule in India and there used to be a British resident in each dominion, ruled by Indian ruler. Nawab Mir Mehboob Ali Khan was the ruler at that time. Surgeon Major Edward Lawrie took charge in 1885 as chief surgeon of Afzalganj hospital as well as Principal Hyderabad Medical School. By his encouragement five lady scholars of Hyderabad joined Medical course. One of them was Miss. Rupa Bai. Lawrie popularised Chloroform anaesthesia and made the medical students proficient in administering it. As a result many students of Hyderabad Medical School participated in animal experiments conducted by the Hyderabad Chloroform Commissions.[2] Sir Thomas Laudor Brunton F.R.S. was the expert from London deputed to Hyderabad by the editor of the British magazine ‘Lancet’ to supervise the proceedings of the second Hyderabad Chloroform Commission in the year 1889 A.D.[3]

**Figure 2:**
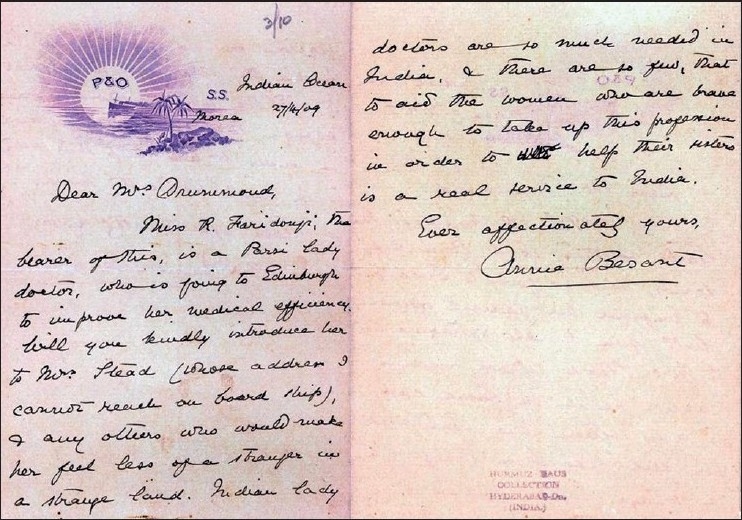
Letter of introduction by Annie Besant

My article on ‘The role of the King Nawab Mir Mehboob Ali Khan convening the two Hyderabad chloroform commissions (1888, 1889 A.D.), at the behest of Lawrie’ was published in 1988 A.D., in local Urdu daily ‘Siasat’. It attracted the attention of Harmusji. He identified Rupa Bai in the group photograph of second Hyderabad Chloroform Commission. He was sportive enough to handover all her certificates and her personal letters to me, to be deposited in the Indian Institute of History of Medicine, Hyderabad. Two letters are of historical importance. Dr. Mrs. Annie Besant, founder President of the Theosophical society of India and Rupa Bai were traveling in the same ship, which sailed from Bombay to Edinburgh. [Figure 2] She wrote a letter of recommendation to Mrs. Drummond dated 27^th^ April, 1909 for Rupa Bai. This handwritten letter is on a letter head of Peninsular and Oriental linear Ship Company. While returning to India from Edinburgh, the ship anchored at Eden port (Saudi Arabia) for few weeks. The British resident wrote a letter to his counterpart in Hyderabad to spare the services of Rupa Bai at Eden as her services are very useful there in Eden. This letter is also available.

## MEDICAL EDUCATION OF RUPA BAI

Rupa Bai completed her studies in Hyderabad Medical School during 1885-1889 A.D. The degree acquired was called as ‘Hakeem’. Now I must explain here, what is this qualification? It sounds like Unani medical qualification. In fact, it was western system of medical education which was imparted in Hyderabad Medical School. This school was started in the year 1846 A.D., by the Muslim ruler of Hyderabad, Nawab Nasirudawla. To start with medium of instruction, the language was by default Urdu because the state language was Urdu. Subjects taught were: Anatomy, Physiology, Materia Medica, Medicine, Surgery and Midwifery. Teachers in the medical school were Englishmen and their lectures were in English language, however, there used to be a Urdu translator. The course was of four-year duration and the examination used to be conducted and supervised by professors of Madras Medical College. But in the year 1885, the medium of instruction was converted to English. It was on account of the efforts made by Lawrie. He was broad-minded in many ways and encouraged the ladies to join the Medical School. He encouraged medical graduates of Hyderabad to go to England and pursue higher medical education. He was responsible for convening two Hyderabad Chloroform Commissions. Two photographs of Hyderabad Chloroform Commissions are available. Both are of second Hyderabad Chloroform Commission. Photograph of First Hyderabad Chloroform Commission is not available. It is amazing to read the anaesthesia case sheets, printed in the book “A report on Hyderabad Chloroform Commissions” published in 1891 A.D. Names of medical students who administered Chloroform anaesthesia have been mentioned.[4,5]

It is remarkable to note here that, in those days, famous medical schools in England and America refused admission to lady candidates. The famous paediatric cardiologist Dr. Miss Taussing of ‘Blalock-Taussing’ surgical technique for ‘Fallots tetralogy’ fame was refused admission in Harvard Medical School of Boston. But John’s Hopkins Hospital, Baltimore permitted Rupa Bai to pursue the medical course.
